# Exploring the Role of Mediterranean and Westernized Diets and Their Main Nutrients in the Modulation of Oxidative Stress in the Placenta: A Narrative Review

**DOI:** 10.3390/antiox12111918

**Published:** 2023-10-26

**Authors:** Cielo García-Montero, Oscar Fraile-Martinez, Diego De Leon-Oliva, Diego Liviu Boaru, Luis M. Garcia-Puente, Juan A. De León-Luis, Coral Bravo, Raul Diaz-Pedrero, Laura Lopez-Gonzalez, Melchor Álvarez-Mon, Natalio García-Honduvilla, Miguel A. Saez, Miguel A. Ortega

**Affiliations:** 1 Department of Medicine and Medical Specialities, Faculty of Medicine and Health Sciences, University of Alcalá, 28801 Alcalá de Henares, Spain; cielo.garcia@edu.uah.es (C.G.-M.); oscar.fraile@edu.uah.es (O.F.-M.); diego.leon@edu.uah.es (D.D.L.-O.); diego.boaru@edu.uah.es (D.L.B.); luism.garcia@edu.uah.es (L.M.G.-P.); melchor.alvarezdemon@uah.es (M.Á.-M.); natalio.garcia@uah.es (N.G.-H.); msaega1@oc.mde.es (M.A.S.); 2 Ramón y Cajal Institute of Sanitary Research (IRYCIS), 28034 Madrid, Spain; raul.diazp@uah.es (R.D.-P.); laura.lgonzalez@uah.es (L.L.-G.); 3 Department of Public and Maternal and Child Health, School of Medicine, Complutense University of Madrid, 28040 Madrid, Spain; jaleon@ucm.es (J.A.D.L.-L.); coral.bravo@ucm.es (C.B.); 4 Department of Obstetrics and Gynecology, University Hospital Gregorio Marañón, 28009 Madrid, Spain; 5 Health Research Institute Gregorio Marañón, 28009 Madrid, Spain; 6 Department of Surgery, Medical and Social Sciences, Faculty of Medicine and Health Sciences, University of Alcalá, 28801 Alcalá de Henares, Spain; 7 Immune System Diseases-Rheumatology and Internal Medicine Service, University Hospital Prince of Asturias, Networking Research Center on for Liver and Digestive Diseases (CIBEREHD), 28806 Alcalá de Henares, Spain; 8 Pathological Anatomy Service, University Hospital Gómez-Ulla, 28806 Alcalá de Henares, Spain

**Keywords:** oxidative stress, placenta, pregnancy, Mediterranean diet, Western diet, high-fat diet, ultra-processed foods, polyphenols

## Abstract

Oxidative stress is a major cellular event that occurs in the placenta, fulfilling critical physiological roles in non-pathological pregnancies. However, exacerbated oxidative stress is a pivotal feature of different obstetric complications, like pre-eclampsia, fetal growth restriction, and other diseases. Compelling evidence supports the relevant role of diet during pregnancy, with pleiotropic consequences for maternal well-being. The present review aims to examine the complex background between oxidative stress and placental development and function in physiological conditions, also intending to understand the relationship between different dietary patterns and the human placenta, particularly how this could influence oxidative stress processes. The effects of Westernized diets (WDs) and high-fat diets (HFDs) rich in ultra-processed foods and different additives are compared with healthy patterns such as a Mediterranean diet (MedDiet) abundant in omega 3 polyunsaturated fatty acids, monounsaturated fatty acids, polyphenols, dietary fiber, and vitamins. Although multiple studies have focused on the role of specific nutrients, mostly in animal models and in vitro, further observational and intervention studies focusing on the placental structure and function in women with different dietary patterns should be conducted to understand the precise influence of diet on this organ.

## 1. Introduction

Oxidative stress is a cellular event that results from an imbalance between free radicals—mainly reactive oxygen species (ROS) and reactive nitrogen species (RNS)—and reduced activity of antioxidant mechanisms [1]. Oxidative stress affects different cell membranes and organelles by structurally and functionally modulating lipids, carbohydrates, proteins, and nucleic acids [2]. Despite abnormal levels of oxidative stress being related to a plethora of disease conditions, a growing body of evidence supports the notion that cells are not simply passive receivers of oxidative stress but that they act dynamically to resist and even to benefit from exposure to oxidants [3]. Indeed, ROS and RNS play roles in several physiological processes, such as the regulation of cell proliferation, differentiation, and death or autophagy [4]. The relevance of ROS and RNS may be due to their crosstalk with several cellular signaling pathways, such as nuclear factor kappa B (NF-κB), MAPKs, Keap1-Nrf2-ARE, PI3K-Akt, Ca^2+^, the mitochondrial permeability transition pore (mPTP), or the ubiquitination/proteasome system [5,6]. However, uncontrolled exposure to environmental stressors like ultraviolet or ionizing radiations, xenobiotics, exacerbated inflammation, hypoxia, and other events leads to a marked dysregulation of oxidative stress, which is related to a broad spectrum of pathologies including cancer, as well as cardiovascular, neurological, and respiratory diseases and rheumatoid arthritis [7]. 

Pregnancy is associated with several changes within a woman’s body. Virtually all organs and tissues of the body undergo significant alterations throughout this period, promoting significant modifications in the cardiovascular, nervous, immune, and endocrine systems [8,9]. Oxidative stress is also increased during gestation, particularly due to systemic inflammation and the aforementioned changes, favoring the physiological adaptations necessary in women for successful pregnancy [10]. However, abnormal levels of oxidative stress during pregnancy are tightly linked to many obstetric disorders [11]. The placenta can be considered a central orchestrator of pregnancy, fulfilling pivotal functions in this period, such as ensuring nutrient support for the fetus while acting as an endocrine organ, immune, and xenobiotic barrier, as well as participating in waste removal [12]. Similarly, a growing number of studies support the relevance of this organ in fetal reprogramming, conditioning newborns to be more susceptible to different diseases in infancy and adulthood [13]. The placenta is a major source of oxidative stress and may favorably influence development and functions or, if exacerbated, promote and enhance pathophysiological conditions affecting this organ, such as pre-eclampsia, miscarriage, and intrauterine growth restriction (IUGR) [14]. All these conditions have significant consequences for the mother and the fetus, and compelling evidence suggests antioxidant-based therapy as a promising area of research for pregnancy success and disease management [15]. Therefore, in the present work, we focus on the relevance of oxidative stress and the critical role of the placenta as a source of ROS and RNS, considering their physiological and pathological consequences. Afterward, the role of diet in the modulation of oxidative stress is considered, focusing not only on antioxidant supplementation but also on lifestyle interventions directed to increase the endogenous antioxidant systems. 

## 2. Development and Cytoarchitecture of the Placenta

The placenta is an organ generated from the embryo after the implantation process. The process by which the placenta is formed and developed is called placentogenesis, which has well-defined stages. First, the implanted embryo (in the form of blastocysts) differentiates into an inner cell mass, which form the fetus, and a trophectoderm, which is responsible for developing the placenta [16]. Prior to implantation, there is another critical process known as decidualization occurs. This consists of a plethora of morphogenetic, biochemical, and vascular remodeling processes of the maternal endometrium surrounding the embryo [12]. In the trophectoderm, there are a special type of cells, i.e., trophoblasts, which are later differentiated into different cells and are responsible for the functions of the placenta [17]. After implantation, syncytial fusion of mononucleated cells leads to the formation of oligonucleated syncytiotrophoblasts (STBs). These cells are responsible for constituting the primary interface in the placenta, separating maternal blood from fetal tissue while ensuring the maternofetal exchange and restricting the entry of harmful substances or immune cells [18]. Beneath the STBs are the cytotrophoblasts (CTBs), which are considered to be stem cells for STBs during villous formation and development [19]. Both STBs and CTBs form the fetal portion of the placenta, known as chorionic villi. However, CTBs are also involved in the invasion of the maternal side of the placenta, forming two types of extravillous trophoblasts (EVTs) known as endovascular trophoblasts (eEVTs), which are involved in the remodeling of uterine spiral arteries and interstitial trophoblasts (iEVTs), which remain in the endometrial decidua and fulfill critical functions in this region [12]. During the first trimester of pregnancy, the blueprint of the placenta is formed by the proliferation and branching of chorionic villi. The initial villi formed by STBs and CTBs (primary villi) are invaded by extraembryonic mesodermal cells, providing a mesenchymal core and converting them into secondary villi [20]. From day 18 to 20, fetal capillaries appear in the core of the secondary villi, leading to the development of tertiary villi. These tertiary villi (also known as mesenchymal villi), despite representing less than 1% of the villous volume at the end of pregnancy, are responsible for the critical endocrine activity of the placenta, leading to the formation of immature intermediate villi and stem villi in the middle of the first trimester [21]. Afterward, stem villi drive to the formation of mature intermediate villi in mid-pregnancy, with a higher degree of fetal vascularization that promotes maternofetal exchange, as well as terminal villi formation, the structure in which diffusive exchange occurs during the late second trimester and the early third trimester [22]. After parturition, the mature placenta is composed of 15 to 28 subunits (cotyledons), which are perfusion chambers irrigated by one or more maternal spiral arteries and partially or entirely isolated from one another by connective tissue [23]. Each cotyledon presents one or more fetal villous tree(s), a fetal artery, and a vein.

**Figure 1:**
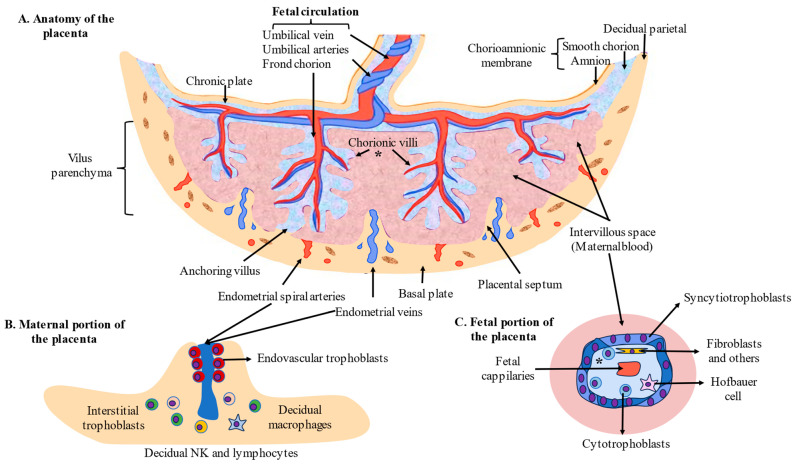
Anatomy and cytoarchitecture of the placenta. (* = General structure of the placental villi).

Finally, in the cytoarchitecture of the placenta, we can distinguish the following parts. (1) In the chorionic villi, STBs are the main cells involved in the maternofetal exchange, whereas CTBs are involved in the regeneration of STBs and EVTs. Fixed and free connective tissue cells originating from the differentiation of mesenchymal cells can also be identified, such as endothelial cells, fibroblasts, myofibroblasts, smooth muscle cells, and macrophages—known as Hofbauer cells—which can also be recruited from maternal circulation [21]. Fetal vessels are equally recognized in the villous trees, comprising capillaries and sinusoids in the terminal villi; arteries, arterioles, veins, and venulas in the stem; and intermediate villi with lumens controlled in an autocrine and paracrine manner [24]. (2) In the maternal portion of the placenta, iEVTs and eEVTs are the identified trophoblastic cells, which are critical for the process of placentation and modulation of different populations of immune cells, mainly decidual natural killers (dNKs), decidual macrophages, and decidual T cells [25]. (3) Fibrinoids, extracellularly deposited materials distributed throughout the placental tissue, comprise two distinctive extracellular matrices: fibrin-type fibrinoids composed of fibrin and maternal blood-clot products that modulate the growth of villous trees and favor blood flow and matrix-type fibrinoids (a product secreted by EVTs that favors trophoblast invasion through specific interactions with cell-surface integrins) [26]. The structure and cells located in the placenta are summarized in Figure 1.

## 3. Oxidative Stress in the Placenta in Physiological and Pathological Pregnancies

Oxidative stress during pregnancy is a double-edged sword for the development of the embryo and the placenta. On the one hand, the placenta continuously generates OS, which contribute to the processes of implantation, placentation, and immunomodulation. On the other hand, uncontrolled oxidative stress in the placenta is a major feature of pregnancy-related disorders, such as pre-eclampsia, intrauterine growth restriction, spontaneous preterm birth, early pregnancy loss, stillbirth, and other pathologies. In this section, we focus on the connection between oxidative stress and the placenta and its implication in physiological and pathological conditions. 

### 3.1. Physiological Role of Oxidative Stress in the Placenta

#### 3.1.1. Origin and Consequences of OS in the Placenta

The mitochondrial free radical theory of aging postulates that ROS harm proteins, macromolecules, and mitochondrial DNA (mtDNA), resulting in respiratory chain malfunction [27]. Increased ROS generation caused by mutated mtDNA facilitates further mtDNA damage and a self-amplifying decline [28]. Because of the increased metabolic activity in placental mitochondria and the increased ROS production brought on by the growing fetus’s higher metabolic demands, pregnancy itself is a state of OS [29]. The placental mitochondria’s production of superoxide anions appears to be a significant source of lipid peroxidation and ROS, which support OS in the placenta. While normal pregnancies maintain a physiological equilibrium between ROS and antioxidant activity, different factors may increase ROS [30]. Under normal pregnancy conditions, the placenta generates ROS from cellular sources, including NADPH oxidase, mitochondrial electron transport, nitric oxide synthase (NOS), and lipids. During healthy pregnancy, the placenta’s oxidants, such as superoxide anion (O_2_) and peroxynitrite (ONOO-), are crucial for trophoblast differentiation and proliferation and placental vascular responses. In mid and late pregnancy, the placenta represents an important source of ROS/RNS production but also contains an array of antioxidant defense systems, receiving some benefits from these molecules and limiting the potential damage associated with oxidative stress [31]. 

Regarding the consequences of oxidative stress in the placenta, it is important to understand that the activation of different transcription factors and the overall context in which oxidative stress acts determine the benefits or harms associated with this event. One of the main determinants of oxidative stress effects in the placenta and different tissues is related to the aforementioned NF-κB. There is a complex relationship between oxidative stress and this transcription factor, as oxidative stress can both activate and inhibit the NF-κB pathway via different mechanisms, and in turn, NF-κB can exert both antioxidant and pro-oxidant actions depending on the context [32]. In the placenta, the activation of this pathway by factors like controlled oxidative stress is responsible for promoting cell migration, invasion, and angiogenesis in healthy pregnancies, whereas under disease conditions, it is associated with enhanced oxidative stress and placental damage [33]. On the other hand, apoptosis in the placenta is a regulated process that increases with placental growth and advanced gestation, although enhanced apoptosis is also observed in different obstetric complications due to different mechanisms of damage like hypoxia and oxidative stress [34]. Again, the activation of certain transcription factors, like forkhead box O (FoxO), induced by OS is responsible for enhancing antioxidant activity and inhibits apoptosis in the placenta; however, when uncontrolled, oxidative stress provides direct effects on different proteins involved in the regulation of apoptosis, which might promote exacerbated apoptosis in pathological conditions [14]. Therefore, not only the sources but also the activation of different downstream products like NF-κB or FOXO in relation to the overall context are essential to understand the relevance and actions of oxidative stress in the placenta.

#### 3.1.2. Implantation and Placentation

Oxidative stress affects the placenta, particularly during early development in the first trimester of pregnancy [35]. Oxidative stress is an important mechanism involved in the implantation process, as the embryo faces a hypoxic environment in the uterus. In particular, oxygen measurements during the first 10 weeks of human pregnancy are reported to be less than 20 mmHg but increase to about 60 mmHg during the second trimester of gestation, gradually declining to 40 mmHg at term [36,37]. Reduced oxygen levels promote ROS/RNS production, and in turn, these molecules activate the main transcription factors involved in the hypoxic response known as hypoxia-inducible factors (HIFs), as well as the NF-κB [38,39]. Collectively, free radicals and HIFs exert important synergic actions, modulating different signaling routes. For instance, hypoxia and oxidative stress synergically favor a change in the embryonic metabolism from oxidative phosphorylation to glycolysis, facilitating implantation, embryo development, and placentation [40]. This condition is sustained through the onset of active placental circulation, approximately at gestational day 12–13. Then, oxidative phosphorylation increases, while glycolysis decreases in the so-called post-implantation embryonic metabolism switch [41]. The process of oxidative phosphorylation performed in the mitochondria is also a critical source of ROS and RNS in different organs, including the placenta [42]. Controlled production of ROS and RNS is critically involved in the angiogenesis and establishment of maternal circulation in the placenta. ROS and HIFs are able to induce the expression of a critical molecule involved in this process, i.e., the vascular endothelial growth factor (VEGF), which is responsible for supporting branching angiogenesis in the first trimester of pregnancy [14]. Not only angiogenesis but also trophoblast invasion seem to be orchestrated by free radicals and the hypoxic environment observed in the first weeks of pregnancy. This occurs through the modulation of various transcription factors involved in placental angiogenesis and trophoblast invasion, such as Krüppel-like factor 8 (KLF8), E26 transformation-specific oncogene homolog (Ets-1), NF-κB, specificity protein 1 (Sp1), Sp3, signal transducer and activator of transcription 3 (STAT-3), and nuclear factor erythroid 2-related factor 2 (Nrf2) [43].

#### 3.1.3. Immunomodulation 

The majority of immunomodulatory actions between maternal immune cells and semiallogeneic fetal cells that prevent immune rejection take place in the placenta. OS also have the ability to modulate certain immune populations in the decidua, such as dNKs, cytotoxic T cells, Tregs, and myeloid-derived suppressor cells (MDSCs) [44], aiding in the proper development of the placenta and fetus. Differential characteristics between dNKs and conventional NKs include elevated CD56 expression and the absence of CD16 (CD56^bright^ CD16^−^). OS could potentially elevate the dNK-to-NK ratio, as dNKs exhibit relative insensitivity to the effects of OS [45]. dNKs present less cytotoxicity but promote fetal tolerance, EVT invasion, and decidual remodeling through the production of several cytokines, such as interferon-gamma (IFN-γ), vascular endothelial growth factor (VEGF), tumor necrosis factor–α (TNF-α), IL-8, and chemokine (C-X-C motif) ligand 10 (CXCL10) [46]. Moreover, killer β-cell immunoglobulin-like receptors (KIRs) are inhibitory receptors that promote immune tolerance. Hypoxia, which increases OS, upregulates KIR expression and the NKG2D ligand and downregulates activation receptors NKG2D, CD3, and CD16 on the NK cell surface, leading to immunosuppression [47]. However, in a state of sustained hypoxia altering the redox state, KIRs are downregulated, and the immune activity of dNKs increases, leading to harmful effects such as excessive apoptosis of trophoblasts, insufficient remodeling of uterine spiral arteries, and blockage of placental circulation [48,49].

ROS produced by monocytes and macrophages also diminish T-cell cytotoxicity through the oxidation of thiol groups on the cell surface, leading to defective signaling and suppression of their activity [50,51]. Lastly, OS can result in the accumulation of immunosuppressive populations, namely Tregs (regulatory T cells) and MDSCs (myeloid-derived suppressor cells), due to their inherent antioxidative capabilities [52,53]. It is noteworthy that MDSCs produce peroxynitrite (ONOO) and nitric oxide (NO), which appear to disrupt T-cell receptor (TCR) signaling through the nitration of both the TCR itself and CD8 molecules [54].

### 3.2. Oxidative Stress in Disease Conditions

#### 3.2.1. Pre-Eclampsia

One of the most studied pathologies affecting the placenta is pre-eclampsia (PE). PE is a systemic disorder belonging to a category of diseases named hypertensive disorders of pregnancy, affecting approximately 3% of pregnancies [55]. The presence of >140 mmHg systolic and >90 mmHg diastolic blood pressure revealed after 20 weeks of gestation constitutes the primary diagnostic criteria for PE. From a clinical standpoint, there are two basic kinds of PE: early-onset pre-eclampsia (EO-PE) and long-onset pre-eclampsia (LO-PE), also known as placental pre-eclampsia [56]. This classification is based on when clinical symptoms first appear, with EO-PE occurring before 34 weeks and LO-PE occurring after 34 weeks. Additionally, the effects of PE on the mother and the fetus, as well as hereditary and clinical characteristics, vary significantly with each manifestation [57]. It is commonly acknowledged that oxidative stress plays a significant role in both PE and placental disorders. PE differs from typical pregnancies in that it is associated with higher levels of oxidative molecules and ROS and lower levels of antioxidant systems [58]. Altered functions of different cells in the placenta are major sources of oxidative cells in PE [59]. PE is linked to increased lipid peroxidation in the placenta and maternal circulation, as well as impaired antioxidant activity. As a result, the spiral arteries are less susceptible to trophoblast invasion, which restricts remodeling to the decidual regions of the arteries and keeps the myometrial segments of the arteries narrow and contractile. As a result, decreased uteroplacental perfusion occurs in PE due to higher vascular resistance in the placenta [60]. Nitric oxide (NO) production is significantly reduced by a decrease in placental endothelial nitric oxide synthase (eNOS) through several mechanisms, making this one of the most significant effects of oxidative stress [60]. In this way, research on women with PE has revealed an inverse relationship between NO levels and levels of sFIt-1 and sEng, indicating that antiangiogenic factors may potentially impede the generation of NO [61]. Both decreased NO synthesis and the decreased bioavailability of its precursor, L-arginine, are seen in PE-affected women [12]. Numerous investigations have found that fetuses with PE, a typical condition linked to aberrant placentation, have considerably higher levels of oxidative stress markers. The levels of particular indicators, like vascular endothelial growth factor (VEGF), soluble fms-like tyrosine kinase (sFIt), and placental growth factor (PIGF), are also altered when pregnant women develop PE [62]. These markers aid in the early diagnosis of disease and the avoidance of serious problems, since they are typically linked to the angiogenic growth of the placenta [63]. These modifications may encourage changes in the renal and cardiac microvasculature, as well as a decrease in the number of embryonic nephrons in vivo, which may also take place in humans [64]. Notably, the utilization of antioxidants such as vitamin C, vitamin E, or n-acetylcysteine has not demonstrated any discernible therapeutic benefits in the management of this condition [65].

#### 3.2.2. Intrauterine Growth Restriction

A common pregnancy issue affecting around 15% of all pregnancies is intrauterine growth restriction (IUGR), a disease in which the fetus does not reach its genetic development potential. IUGR increases the risk of fetal mobility and death, as well as susceptibility to cardiovascular disease later in life [66]. Approximately 76% of intrauterine deaths are linked to IUGR [67].

Malondialdehyde (MDA), a byproduct of fatty acid oxidation, is routinely quantified in plasma and tissue samples to assess lipid peroxidation and OS. When compared to healthy pregnancies, patients with IUGR pregnancy have higher levels of MDA and xanthine oxidase in their placental tissues, umbilical cord plasma, and maternal blood, which imply the involvement of OS in IUGR [68,69]. Moreover, in placentas with IUGR, the mRNA levels of the glutaredoxin and thioredoxin reducing systems are also decreased. In addition, an IUGR placenta exhibits age-related indicators such as shortened telomeres and decreased telomerase activity [69]. In placentas from IUGR pregnancies, a significantly shorter telomere and/or absent or reduced telomerase activity are seen, with a lower expression level of hTERT [70]. Additionally, telomere-induced senescence markers p21 and p16 are expressed more, while antiapoptotic protein Bcl-2 is diminished in IUGR placentas. The data on aging markers support the idea that oxidative stress plays a role in placental aging and IUGR, together with increased oxidative stress indicators and decreased antioxidant capacity [71].

#### 3.2.3. Gestational Diabetes Mellitus

Gestational diabetes mellitus (GDM) represents one of the most common complications of pregnancy, as defined by the emergence of chronic hyperglycemia during pregnancy without a prior diabetes diagnosis [72]. The prevalence and incidence of this condition are rising worldwide [73], closely linked to obesity, sedentary lifestyle, multiparity, family history of type 2 diabetes mellitus (T2DM), previous history of GDM or the delivery of a macrosomic infant, advanced age of the mother, ethnicity, and polycystic ovarian syndrome [74,75]. Pathophysiologically, an inefficient adaptative response of the pancreas during pregnancy, combined with poor insulin sensitivity (probably impaired prior to pregnancy), is responsible for driving β pancreatic cell dysfunction and hyperglycemia, affecting different organs, like the placenta [76]. In turn, placental changes also participate in the pathophysiology of GDM, mainly by releasing different products that enhance and exacerbate impaired insulin sensitivity and hyperglycemia [77].

Oxidative stress in different organs has also been noted as a critical mechanism of GDM [76]. Indeed, enhanced oxidative stress and inflammation in the placenta have been observed in women with GDM [78]. Among other markers, two-fold increases in placental release of 8-isoprostane, lipoperoxidases, and protein carbonyl contents have been observed in placentas obtained from women with GDM, together with reduced enzymatic activities of SOD and catalase antioxidants [79,80]. Therefore, the use of antioxidants and strategies aimed to ameliorate placental and fetal oxidative stress is currently being investigated as a promising strategy in GDM, although further efforts are still warranted [81].

#### 3.2.4. Spontaneous Preterm Birth

Preterm birth (PTB) is characterized by the occurrence of childbirth prior to the completion of 37 weeks of gestation. PTB was estimated by the World Health Organization (WHO) to account for 9.6% of all births in 2005 [82]. Unknown causes of spontaneous PTB are the most prevalent manifestation of PTB. A premature preterm rupture of the fetal membrane (pPROM), which occurs 30–40% of the time before spontaneous PTBs, is thought to account for about 60% of PTBs [83]. Although these women have a lower PTB prevalence (3–5%), they account for the majority of clinical practice. 

Inflammation plays a significant role in the pathophysiology of PTB, and multiple biomarkers have been linked to worse outcomes [84]. It is interesting to note that early pPROM (34 weeks) has shorter telomeres than intact PTB membranes that are gestationally age-matched. However, early pPROMs and term deliveries (>40 weeks) were found to have similar telomere lengths, suggesting a connection between the two pregnancy phenotypes [85]. It is suggested that pPROM may include early-aging fetal tissues that cause rupture and delivery, owing to the several similarities in molecular and histologic markers between pPROM and normal-term births.

#### 3.2.5. Early Pregnancy Loss and Stillbirth

A pregnancy loss is, by definition, any pregnancy loss that occurs between the time of conception and 24 weeks of gestation or before the fetus achieves viability—whichever comes first. A proportion of 0.5–2% of women of childbearing age experience recurring pregnancy loss (RPL), which is characterized as three or more consecutive miscarriages. The European Society of Human Reproduction and Embryology (ESHRE) produced a consensus statement that defines recurrent pregnancy loss as two or more pregnancy losses that cab be identified by serum or urine human chorionic gonadotropin [86]. One of the major issues in the field of reproductive medicine is RPL, which causes mental stress for couples who are impacted. Recurring pregnancy loss is largely caused by a combination of illnesses, including advanced maternal age, as well as the gestational age of the last pregnancy that ended in loss and its underlying etiology [87,88]. Cellular oxidants play a role in preserving the redox equilibrium required for the reproductive health of typical women and the success of their pregnancies [89]. The particular mechanism of this interaction is still largely unknown, although the data that are now available support the idea that oxidative stress and OS-mediated damage are involved as important elements in the etiology of RPL [90]. Smoking and alcohol use are significant contributors to oxidative stress and have the potential to have a severe influence on the health of the mother and fetus [89].

Another serious obstetric complication is stillbirth, which is defined as intrauterine fetal death at or after 24 weeks of gestation [91]. Although a variety of risk factors for this complication have been identified, such as advanced maternal age, obesity, smoking, late gestational age, and IUGR, the majority of cases remain unexplained [92]. Recent investigations of stillbirth have suggested a link between placental pathologies, such as infarction, artery wall thickening, calcification, and malfunction [93]. However, one study published in 2016 reported telomere-dependent senescence in the placenta, indicating a significant drop in telomere length in placentas linked with unexplained stillbirth, which may result in premature placental aging and placental dysfunction, leading to fetal mortality [94,95]. In this sense, oxidative stress, which causes single and double DNA strand breaks, accelerates telomere shortening.

#### 3.2.6. Excessive Gestational Weight Gain

Excessive gestational weight gain (EGWG) is a growing condition associated with potential harmful effects such as hypertensive disorders of pregnancy, fetal macrosomia, and increased cesarean birth rates [96]. Previous works have identified individual, family, and social factors related to this condition, such as prepregnancy overweight (including obesity), younger age (≤30 years old), unemployed status, primiparity, smoking, and being unmarried (including divorced) [97]. The placenta also plays a significant role in women who suffer from EGWG, as previous works have evidenced that a lowered fetal birth weight/placental weight can be observed in this group, even when compared to pregestational obese mothers [98]. In addition, systemic oxidative stress seems to be tightly linked to EGWG, specially due to abnormal adipose tissue accumulation [99]. In parallel, the placentas of these women also present enhanced oxidative stress, as defined by an increased presence of carbonyl proteins; elevated activity of NOX; and a decrease in SOD and other antioxidant defense activities [100]. While additional efforts are needed, placental oxidative stress also seems to be a consequence of and cause the exacerbation of damage associated with EGWG. 

#### 3.2.7. Chronic Venous Disease

Chronic venous disease (CVD) refers to a group of venous disorders that generally involve lower-extremity edema, trophic skin changes, and discomfort resulting from elevated venous pressure [101]. During pregnancy, a set of hemodynamical and cardiovascular changes takes place, increasing the risk of suffering from CVD, especially in the third trimester. Our investigations have revealed increases in oxidative stress and inflammatory markers in the placenta, umbilical cord, and blood of pregnant women with CVD [102,103,104]. Using RT-qPCR and immunohistochemistry, we determined an increase in oxidative stress markers NOX1, NOX2, iNOS, PARP, and ERK in the placental tissue of women with CVD in comparison to healthy pregnant women, as well as malondialdehyde (MDA) plasma levels [104]. Transcription factor NRF2 is critical in the regulation of the response to oxidative stress. NRF2 is also upregulated in the placental tissue of women with CVD, while Keap1, CUL-3, and GSK-3β gene and protein expressions were found to be significantly downregulated [102]. Therefore, it is hypothesized that CVD during pregnancy is associated with placental dysfunction, with oxidative stress reported as a pathophysiological mechanism in these studies.

#### 3.2.8. First-Episode Psychosis

**Figure 2:**
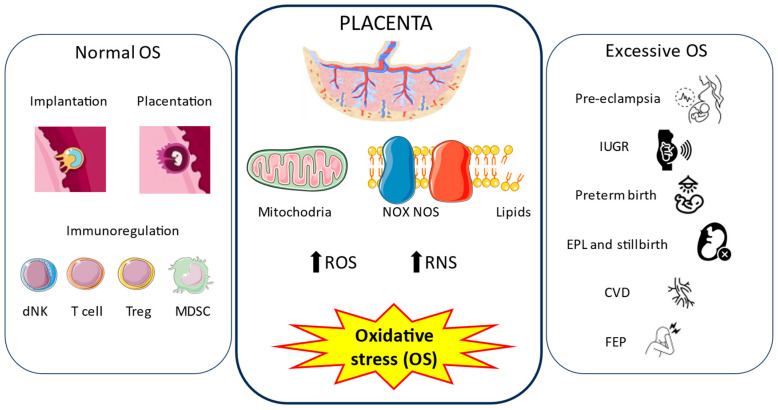
Oxidative stress in the placenta. During pregnancy, the placenta continuously generates oxidative stress (OS) through the respiratory mitochondrial chain, NADPH oxidase (NOS) and nitric oxide synthase (NOS) enzymes, and the peroxidation of lipids. These lead to an increase in the amount of free radicals, mainly reactive oxygen species (ROS) and reactive nitrogen species (RNS). Normal levels of OS play important roles in implantation, placentation, and immunoregulation, which drive a healthy pregnancy. However, excessive production of free radicals is correlated with different pregnancy-related disorders, such as pre-eclampsia (PE), intrauterine growth restriction (IUGR), preterm birth, early pregnancy loss (EPL) and stillbirth, chronic venous disease (CVD), and first-episode psychosis (FEP) during pregnancy.

Psychosis is a complex psychiatric disorder characterized by a loss of touch with reality, comprising delusions, hallucinations, and disorganized thought and behavior [105]. Although first-episode psychosis during pregnancy is a rare event, it is associated with adverse obstetric and neonatal outcomes such as cesarean delivery, impaired fetal growth, placental abruption, antepartum or postpartum hemorrhage, fetal anomalies, fetal distress, and stillbirth [106]. We previously demonstrated significant histopathological changes in the placentas of women who suffered first-episode psychosis (FEP) during pregnancy, such as an increase in the number of placental villi, bridges, syncytial knots, and syncytial knots/villi, as well as hypoxic marker HIF-1α and apoptotic markers BAX and Bcl-2 [107]. Moreover, the gene and protein expressions of oxidative stress markers NOX-1, NOX-2, iNOS, and PARP were found to be upregulated, suggesting a direct link between FEP and oxidative stress in the placenta [103]. In parallel with this fact, we observed an increase in lipid peroxidation (MDA levels) and ferroptosis, a special type of cell death linked with ROS generation and oxidative stress [108]. However, the origin and role of OS are not clear. A possible link could be the different changes occurring in this organ, such as hypoxia, as previously mentioned, together with exacerbated oxytocin expression in this tissue observed in pregnant women [109]. Further studies are needed to deepen understanding of this complex association. Figure 2 summarizes the central role of oxidative stress in the placenta in physiological and pathological pregnancies explained in this section.

## 4. Maternal Diet, Oxidative Stress, and the Placenta Connection and Translational Opportunities

Diet is a critical factor during pregnancy, exerting pleiotropic functions that aid in the proper development of this period and contributing to maternofetal well-being [110]. However, nutritional recommendations of healthy dietary patterns that include nutrient-dense whole foods; fruits; vegetables; legumes; whole grains; healthy fats with omega 3 fatty acids that include nuts and seeds; and fish instead of poorer-quality, highly processed foods are often unfollowed [111]. Healthy foods and dietary patterns such as a Mediterranean diet (MedDiet) provide multiple benefits in the organism and are even able to ameliorate oxidative-stress-induced diseases [112]. Conversely, unhealthy foods and dietary patterns such as those included in Westernized diets (WD) are known to induce systemic inflammation, gut dysbiosis, and other mechanisms closely linked to oxidative stress [113]. A growing body of evidence supports the relevance of diet during pregnancy in terms of placental composition and function [114,115,116]. Indeed, compelling evidence supports the efficacy of nutritional interventions and supplementation in managing placental complications, although further studies are warranted in this sense [117]. In this section, we explore the relevance of a MedDiet and its main nutrients in the placenta of pregnant women in comparison with the effects of a WD and unhealthy patterns in the same organ.

### 4.1. Role of Mediterranean Diet and Related Nutrients and Oxidative Stress in the Placenta 

A MedDiet is a healthy dietary pattern with considerable reported evidence. This diet is characterized by a combination of highly complex carbohydrates in fiber (obtained from vegetables, fruits, cereals, and legumes), polyunsaturated and monounsaturated fatty acids (present in extra virgin olive oil, fish, seeds, and nuts), and different micronutrients and bioactive compounds derived from plant-based foods with critical antioxidative properties, emphasizing the role of polyphenols [118]. Previous works have found an association between adherence to a MedDiet and positive outcomes in pregnancy, such as reduced risk of suffering gestational diabetes mellitus, as well as limited weight gain and favorable consequences for the offspring [119]. This diet also has notable benefits for the placenta. Indeed, maternal diet before conception and during pregnancy has been demonstrated to exert significant effects on placental development [119,120]. Likewise, a recent study showed that exercise training in combination with a MedDiet seems to reduce telomere shortening and aging in this tissue [121], whereas different foods and nutrients contained in a MedDiet are able to improve placental flow [122]. As the precise role of a MedDiet in placental structure and function remains to be fully characterized, we focus on the role of the main nutrients contained in this diet (polyphenols, fiber, omega 3, choline, and vitamins) in the placenta, with a special focus on oxidative stress modulation during pregnancy.

#### 4.1.1. Fiber and Polyphenols

Polyphenols and dietary fiber constitute a group of compounds found in plant-based foods, mainly fruit, vegetables, and spices. Polyphenols are mainly classified according to their chemical composition in four main families: flavonoids, lignans, stilbenes, and phenolic acids [123]. These compounds have many favorable effects on human health, presenting antioxidant, cytotoxic, anti-inflammatory, antihypertensive, and antidiabetic activities, as well as exerting notable effects on the gut microbiota [124]. In pregnancy, polyphenols have the potential to ameliorate oxidative stress and inflammation, exerting multiple pleiotropic actions [125]. No minimum recommended doses of polyphenol intake have been established to date, either in pregnant or normal subjects [126,127]. According to past works, pregnant women tend to consume more polyphenols daily than adult women and girls, with an average intake of 2064 mg/day [128]. However, excessive polyphenol intake at early stages of pregnancy could also have detrimental consequences, impairing placental and fetal development [129]. Thus, it is advisable not to include supplementation with polyphenols in the first trimester of pregnancy, although the intake of polyphenols contained in fruits and vegetables does not seem to have negative effects and may be included as part of a healthy and balanced diet in pregnant women. Despite this consideration, translational applications of polyphenols to improve placental function in different models of disease are being increasingly explored. For instance, it has been demonstrated that polyphenols are able to improve the transport of different bioactive compounds in the placenta; however, their effects seem to depend on the exposure and combination with other polyphenols [130].

Flavonoids are the predominant type of polyphenols and can be subdivided into anthocyanins, chalcones, dihydrochalcones, dihydroflavonols, flavanols, flavanones, flavones, flavonols, and isoflavonoids [129]. As an example of flavonoids, quercetin and kaempferol are two types of flavonols that can cross the placenta during pregnancy and accumulate in the fetus [131,132]. Quercetin is mostly found in onions, grapes, berries, cherries, broccoli, and citrus fruits [133], whereas kaempferol is particularly abundant in leafy-green vegetables like spinach, kale, Chinese cabbage, and spices (dill) [134]. Animal models of pre-eclampsia have shown that both quercetin and kaempferol can be used to alleviate hypoxia and promote angiogenesis through the modulation of HIF-1 [135]. In a similar manner, quercetin can suppress the production of TNF α, IL-6, and MCP-1 in the placenta and downregulate the plasma level of inflammatory molecules while ameliorating oxidative stress [136], promoting favorable changes in the histological structure and function of the placenta in rats with gestational diabetes mellitus [137]. Also, both quercetin and hesperidin (a type of flavanone) and their metabolites seem to provide beneficial effects alone or in combination on trophoblast cell lines against hypoxia reoxygenation-induced oxidative stress by enhancing GSH levels and inhibiting p38, MAPK, and JNK activation [138].

Other important polyphenols that have been studied include those contained in tea leaves and coffee, such as epigallocatechin gallate (EGCG) in the former and caffeic acid (CA). EGCG is a type of flavanol whose relevance in the placenta is receiving growing attention. In a recent study, Almeida-Toledano et al. [139] found that this polyphenol is able to ameliorate alcohol-induced placental changes in angiogenic factors and oxidative stress, demonstrating the potential role of this compound in limiting the fetal alcohol spectrum disorder phenotype and related consequences. On the other hand, CA is a type of phenolic acid that seems to have a favorable effect in the alleviation of oxidative stress in human trophoblast cell lines, probably by stimulating the antioxidant systems (GSH) and participating in effective ROS scavenging [140]. Resveratrol, the main type of stilbene mainly found in red grapes, also provides multiple antioxidant and anti-inflammatory effects in the placenta. Thus, this polyphenol has been demonstrated to be capable of protecting human trophoblasts against H_2_O_2_-induced oxidative stress by activating sirtuin-1 (SIRT1)-dependent autophagy [141]. Another study [142] showed that resveratrol (50 μmol/L) treatment for 8 h can improve the detrimental effects caused by H_2_O_2_ in HTR-8/SVneo cells, favoring cell survival, reducing MDA levels, and increasing the levels of SOD and CAT. Finally, curcumin is another polyphenol from the curcuminoid family that protects against H_2_O_2_-induced oxidative stress through the limitation of apoptotic cell death and the activation of the Nrf2 signaling pathway [143]. Overall, the promising role of polyphenols in favorably modulating placental function and reducing pathological oxidative stress is supported by multiple studies. However, different questions, such as the dose and the moment during pregnancy at which polyphenols must be considered, remain to be answered, as limiting oxidative stress in early stages could have negative consequences. Furthermore, the polyphenols that are most commonly consumed in our diets are not necessarily the most bioavailable due to inefficient absorption or rapid excretion, as the chemical structure of polyphenols (not their concentration) influences the rate and extent of absorption and the nature of the metabolites circulating in the plasma [129]. However, due to the complete food matrix that plant-based foods present, with multiple beneficial compounds that interact and exert positive synergic actions, foods rich in different types of polyphenols should be included, and their nutraceutical use and applications should be deeply explored in parallel.

Dietary fiber (DF) is composed of a set of non-digestible carbohydrates that exert beneficial effects in the gastrointestinal tract and includes a broad spectrum of substances, like non-starch polysaccharides, cellulose, pectins, inulin hydrocolloids, fructo-oligosaccharides, and resistant starch [144]. According to previous works, dietary intake of DF by pregnant women in the United States tends to be low (17.3 g/day), whereas the minimum recommended dose is around 28 g/day [145]. In other studies, only a small percentage of pregnant women (29.5%) reached the minimum recommended dose of DF through increased inclusion of fruits and vegetables [146]. Excessive intake of DF is rare but is associated with impaired absorption of nutrients and diarrhea during pregnancy [147]. The use of DF supplementation in pregnancy has been demonstrated to improve insulin sensitivity, modulate the gut microbiota and metabolites, and increase placental vascular density [148]. Previous works have evidenced that DF can raise placental serotonin levels by promoting maternal serotonin synthesis in the colon and transport from the mother to the placenta [149]. Simultaneously, DF consumption has been demonstrated to enhance maternal, placental, and fetal antioxidant defense capacities [150]. For instance, maternal long-term inulin intake improves the placental redox status and nutrient transport, mainly through favorable effects on the gut microbiota and metabolites [151]. Lin et al. [152] demonstrated that DF supplementation is superior to N-acetylcysteine in limiting maternal serum and placental superoxide anion and hydroxyl radical scavenging, acting as an important antioxidant mechanism on the placenta. Overall, the inclusion of DF and polyphenols mainly contained in foods has the potential to alleviate oxidative stress in the placenta and other tissues, whereas many questions remain with respect to adequate doses for each condition.

#### 4.1.2. Vitamins

Supported by animal models and clinical observations of outcomes associated with various placental pathologies, we suggest that unresolved vitamin deficiencies pose a potential danger to developing humans, even in their adolescence and in adult life. The supplementation of some vitamins has been studied in order to meet the mother’s nutritional requirements and prevent derivative complications, such as physical, skeletal, immune, neurological, and cognitive impairment [153]. According to recommendations of experts for pregnant women, minimum daily intake of vitamins A (800 μg), B1 (1.4 mg), B2 (1.4 mg), B3 (18 mg), B6 (1.9 mg), B9 (400 μg), B12 (2.6 mg), C (70 mg), D (200 IU), and E (10 mg) should be provided [154]. The most commonly observed vitamin deficiencies in pregnant women are liposoluble vitamins D (with important immune function), A, and E, as well as hydrosoluble vitamins from B complexes like B12. Conversely, under supplementations, excessive consumption of other vitamins, like vitamins K and B9, can also been observed [155].

Vitamin D deficiency in pregnancy is the most studied and monitored type of deficiency. In fact, it is considered an important risk factor for poor fetal and neonatal development, as well as rickets in infancy [156]. In this context, immunohistochemical, Western blotting, and qRT-PCR assays identified enhanced oxidative stress in placentas from rats with 25-hydroxyvitamin D deficiency. The offspring were studied at weeks 0 and 16 and presented altered expression levels of Nrf2, CBR1, and inflammatory cytokines (IL-1β, IL-6, and TNF-α) in the liver and pancreas. This dysregulation led to a metabolic syndrome in both mothers and their offspring [157]. Gestational diabetes and pre-eclampsia animal models show significantly disrupted vitamin D signaling and calcium transport [158]. Supplementation has been contemplated for these gestational complications, as maternal vitamin D is the only source for the future neonate. Deficiencies in the mother have also been related to increased rates of caesarean delivery, low birth weight or small for gestational age, allergies, and respiratory conditions [153]. Vitamin D deficiency is also associated hyperhomocysteinemia. The assessment of these vitamins may be key in inferring the production of oxidative stress markers. In particular, vitamin D may target oxidative-stress-induced COX-2 upregulation and thromboxane release. At the same time, the n-3/n-6 fatty acid ratio can benefit from micronutrient supplementation [159].

Conversely, vitamin E deficiency in pregnancy has been suggested to be linked to placental aging, vascular endothelial injury, hypertension, miscarriage, and premature birth. However, it is a less common nutrient measured in pregnant women [160]. Rat pups supplemented with vitamin E showed increased antioxidant activity in plasma. Researchers also found that some optimized formulas with enhanced bioavailability could achieve better results [161]. Moreover, a study conducted by Johnston et al. aimed to prevent pre-eclampsia in type 1 diabetic women, given that placental oxidative status is greater in this condition. Supplementation with vitamins C and E was not found to modulate placental antioxidant enzymes (GPx and glutathione reductase (Gred)), although the histological architecture was not contemplated like in other studies [162]. Nevertheless, treatment is commonly found to be time-limited in such human intervention studies.

B-complex vitamins like B12 are associated with oxidative-stress-related factors like hyperhomocysteinemia, which is a risk factor for preterm birth and low birth weight. High MDA levels seem to be correlated with B12 deficiency, as well as increases in other proinflammatory cytokines (TNF-α), angiogenesis factors (VEGF-A), and metalloproteinases (MMP2 and MMP9) [163]. No significant animal models or controlled trials assessing B12 supplementation in the context of oxidative stress pathways in the placenta environment are currently available.

Supplementation with folic acid and vitamin B9 has been found to be effective in pregnancy. In particular, high-fat feeding rat models have demonstrated similar outcomes to those reported in previous studies. Folic acid supplementation was found to attenuate the consequences of an unhealthy diet, with an increase in the expression of Nrf2 and SIRT1 and downregulation of NF-κB. Other investigated effects included the alleviation of angiogenesis factors, blood sinusoid area, and damage to vascular density caused by diet-induced placental vascular dysplasia [164].

Finally, although vitamin C is not a common deficiency, its addition to some diets is interesting owing to its antioxidant power. In a cohort of 200 pregnant women with gestational diabetes mellitus, a randomized controlled trial was conducted, which involved the administration L-ascorbic acid or vitamin C to the treatment group. The results showed that the use of this antioxidant can significantly reverse effects of oxidative stress in women and lower levels of superoxide dismutase (SOD), catalase (CAT), and glutathione peroxidase (GPx) enzymes, as well as glutathione (GSH) and malondialdehyde (MDA). With respect to the metabolic health of the offspring, improved blood sugar levels and lower NICU (Neonatal Intensive Care Unit) admission rates were observed [165]. 

Another nutraceutical of interest is tetraterpene carotene lycopene, which is a type of provitamin A. An animal model of high-fat-fed rats during pregnancy revealed that the addition of this compound can reduce the negative effects of this diet on the placenta through the IL-17 pathway, increasing GPx and total antioxidant capacity and decreasing ROS. The results in the offspring also seemed positive, with an increased average fetal weight compared to untreated rats [166].

#### 4.1.3. Polyunsaturated Fatty Acids

Finally, in the case of essential fatty acids (FAs), given the link between their deficiency and the increased production of ROS, we can also find studies related to the oxidative status of the placenta. Considering that the World Health Organization (WHO) and the Food and Agriculture Organization of the United Nations (FAO) recommend daily intake of n-3 PUFAs like n-3 docosahexaenoic acid (DHA) for improved quality of gestation [167], this group of nutrients should be monitored with respect to oxidative-stress-related placental pathologies. Apart from DHA, other types of n-3 PUFAs include alpha-linolenic acid (ALA) and eicosapentaenoic acid (EPA). Both DHA and EPA are mainly obtained from fish oil and fatty fish like salmon or tuna, whereas ALA is more commonly found in seeds and nuts. Notwithstanding the fact that ALA can transform into DHA and EPA, its conversion rate is very low, evidencing the need to include both fish and plant-based foods to ensure an adequate intake of n-3 PUFAs [168]. To date, there is not a clear consensus on recommended dietary allowance (RDA) for n-3 PUFAs across all lifespans, including pregnancy. However, adequate intake of ALA in pregnancy seems to have been established at 1.4 g/day, whereas adequate intake of EPA and DHA corresponds to 350–450 mg/day, either through the inclusion of foods rich in these nutrients or via supplementation [169].

After the administration of n-3 PUFAs, in vitro immunofluorescence and mRNA expression studies have shown increased expression of antioxidant-related genes like Sirt1 and Bcra1/Msh2, which are key in DNA repair [170]. These FA nutrient effects have already been widely studied in numerous pathologies, like metabolic syndrome, cardiovascular disease [171], chronic obstructive pulmonary disease (COPD) [172], pancreatic problems [173], and endocrinal and sexual conditions like polycystic ovaries [170], among others. One limitation noted in our literature search is that placental oxidative stress conditions has been less explored; therefore, future clinical trials should rely on available data from animal models.

Low levels of omega 3 (n-3) poly and monounsaturated fatty acids (MUFAs and PUFAs, respectively) have been found in women with pathologies like pre-eclampsia. The rat model reported by Joshi included supplementation with n-3 FA and vitamin E, resulting in the amelioration of late-onset but not-early onset pre-eclampsia and certain factors related to oxidative stress, like peroxisome proliferator-activated receptor gamma (PPAR-g) or VEGF, both of which are relevant in angiogenesis [174]. The same combination of supplements applied to a rat model by Kasture et al. resulted in reduced apoptosis (lower levels of caspases 8 and 3) in pre-eclamptic placentas [175].

Mezouar et al. cultured T1D placenta extracts with a combination of vitamins C and E, as well as FAs n-3 and n-6 PUFA and n-9 MUFA [176]. Their results showed increased glucose uptake, MDA levels, and basal cell proliferation but reduced CAT activity and glutathione concentrations in diabetic patients compared to controls. With the addition of supplements, they found improved redox status and cell function in diabetic samples compared to untreated pathological. These results were not achieved totally when vitamins and FAs were administered separately [176].

Another in vitro study conducted by Melody et al. involved the used of an explant model of placentas at term in patients who had consumed fish or fish oil during the month prior to delivery. Thei aim was to find a differential expression of proinflammatory cytokines, finding reduced placental IL-6 when taking n-3 but a contrary effect when they had taken a more complex mix of PUFAs. The interpretation of these results is subject to the limited time of exposure and the small sample size of the study cohort [177]. Another mouse model provided controversial results, suggesting that the consumption of fish oil before and during gestation may increase LPS-induced inflammation in the placenta, amniotic liquid, and uterus [178]. Other animal models have shown that maternal dietary addition of n-3 PUFAs improves fetal growth and reduces oxidative status in the placenta [179]. Related to fetal growth restriction, a different study of ischemia–reperfusion in a pregnant rat model also investigated oxidative stress pathways. After 22 days of n-3 PUFA supplementation, the activity of cytosolic superoxide dismutase (SOD) was increased significatively, with no evidence of protection against growth restriction related to ischemia–reperfusion injury [180]. Other animal models have focused on the prevention of preterm labor, including n-3 PUFAs in the diet of pregnant animals due to their properties that contribute to the fluidity and permeability of cell membranes. 

Interestingly, the supplementation of these FAs can ameliorate the consequences of other micronutrient deficiencies. For example, the addition of n-3 FA and folic acid in a rat model of vitamin B12 deficiency was found to ameliorate the expression of PPARg, MDA, and placental TNF-α and IL-6 [181].

Once again, unity makes strength. The combination of PUFAs with antioxidant vitamins (E) and minerals (selenium) in a high-bioavailability matrix like enriched hen egg is of interest in terms of prompting anti-inflammatory conditions, as proven by Susnjara et al. in controlled trials with healthy subjects [182]. All these results are promising although perhaps difficult to reproduce. However, these findings at different levels of the oxidative stress pathway are worth considering in wider and deeper studies of pregnant women.

#### 4.1.4. Choline

Choline is an essential nutrient required for structural integrity and signaling functions of cell membranes, as well as neurotransmission and muscle and hepatic functions, in addition to acting as the major source of methyl groups in the diet [183]. This nutrient also provides significant benefits during pregnancy, with daily recommended intake of 450 mg/day [154]. However, a significant percentage of pregnant women do not achieve the minimum recommended dose of this nutrient, which is mainly found in animal-based foods (beef, eggs, chicken, fish, and pork, providing more than 60 mg per 100 g); plant-based foods (nuts, legumes, and cruciferous vegetables, providing at least 25 mg per 100 g); and, despite not being especially enriched in this nutrient, cow milk, which can also represent an important source of choline [184]. The placenta can participate in the metabolism of choline and transform this nutrient into acetylcholine to be used by the fetus [184]. Choline also provides multiple benefits in the placenta, as evidenced in prior studies. For instance, this nutrient is able to directly change the placental epigenome in murine models through several mechanisms [185]. This epigenetic action can provide significant effects in the offspring, as previous works conducted in humans have demonstrated that increased choline intake in the third trimester of pregnancy can modulate the expression of critical genes involved in the hypothalamic–pituitary brain axis, potentially determining the stress response in the fetus [186]. This nutrient also has notable benefits in terms of placental function, modulating angiogenesis, inflammation, and macronutrient transport [184]. Interestingly, increased intake of choline with supplementation (930 mg/day) was able to reduce the levels of a critical antiangiogenic factor involved in pre-eclampsia (Sflt-1) when compared to the recommended intake (450 mg/day), suggesting that increased doses of choline can be used to treat certain placental pathologies [187]. Likewise, choline is able to mitigate oxidative stress and apoptosis in throphoblasts, demonstrating the potential of this nutrient to improve placental insufficiency [188]. Additional studies are needed to deepen understanding of the precise role of choline in the placentas of women during healthy and pathological pregnancies, but compelling evidence supports the need for this nutrient to be strongly considered for maternofetal well-being.

### 4.2. Role of Westernized and Unhealthy Dietary Patterns in the Placenta

The impact of WD and other models of unhealthy diets, such as a high-fat diet (HFD), on the placenta has been previously studied. WD is characterized by the inclusion of ultra-processed foods and drinks (UPFDs), refined grains, processed meat, sweets, candies, high-sugar beverages, fried foods, high-fat dairy products, and high-fructose products [189]. HFDs are defined by patterns under which lipids account for more than 30% of the total energy intake [190]. The effects of an HFD on health needs to be elucidated. On the one hand, the proportion of fat in the diet does not appear to be as crucial as its combination with other nutrients and food sources from which it originates. Thus, an HFD that includes a high quantity of carbohydrates (specially refined sugars) has more detrimental effects than when these are present in a lower proportion [191]. Also, while saturated fats have traditionally been at the center of criticism, current evidence supports the notion that the unhealthiest fats are trans fats primarily derived through the refinement of ultra-processed foods. Likewise, polyunsaturated fats from refined oils may also lead to adverse health outcomes, whereas saturated fats from unprocessed animal-derived foods may exert more beneficial than harmful effects on health [192].

The roles of the WD and HFDs are receiving increasing attention in pregnant women. On the one hand, both WDs and HFDs during pregnancy have been associated with detrimental outcomes for both the mother and fetus [193,194]. Regarding the effects of these diets on the placenta, previous works have shown that HFDs are able to induce significant alterations in the placental structure and metabolism [195]. In particular, 8-week-old female rats fed this type of diet during pregnancy showed significant epigenetic changes, including reduced levels of free fatty acid receptor 3 (FFAR-3), and exhibited enhanced oxidative stress. Similarly, a high-fat and -sugar diet administered 6 weeks prior to and during pregnancy in a female mouse model promoted oxidative stress in the placenta, which was accompanied by abnormal placental function and marked reductions in placental weight, surface area, and maternal blood spaces [196]. It seems that time-restricted feeding (TRF) can limit placental inflammation and oxidative stress related to HFDs [197], suggesting the cumulative effects of this diet in placental damage. Also, non-human primates models show that WDs are associated with enhanced inflammation and oxidative stress in the placenta and fetus, especially through the modulation of various micro-RNAs (miRNAs) like miR-1285-3p, which are involved in the modulation of NFE2L2, am oxidative stress marker altered in the placenta under disease conditions [102]. Other studies have failed to find an association between HFD and oxidative stress in the placenta, demonstrating that future studies should deeply explore the connection between different dietary patterns and oxidative stress in this tissue [198].

Many effects of artificial additives on the human body, especially during placentation, are still unknown. What we have recently learned about sweeteners from in vitro and in vivo models is that their metabolism manifests toxicity. One study found that aspartame consumption during pregnancy may impact the structure, growth, and function of the placenta through sweet taste receptor-mediated stimulation of oxidative stress. Pregnant mice treated with aspartame had lower fasting blood glucose levels, elevated systolic blood pressure, and reduced placental and fetal weights. The study also revealed that aspartame metabolite phenylalanine also induced ROS production and affected trophoblast proliferation. These ROS hyperactivate Akt and downregulate mitochondrial antioxidant manganese superoxide dismutase (MnSOD) [199]. In another in vitro study, researchers showed that aspartame also had an antiproliferative effect in extravillous trophoblasts, resulting in reductions in metabolically active cells, glucose uptake, and cell protein content, as well as an increase in cells arrested in the S phase [200]. In another study, the in vitro exposure of human trophoblasts to D-galactose resulted in less activity and premature senescence via the SIRT1/FOX3a/ROS signaling pathways [201]. These studies support the dangers of the consumption of sweeteners and other additives for placentation processes. They also suggest the possibility of oxidative stress damage and premature aging caused by sweeteners.

Finally, previous works have found a direct association between excessive sodium intake observed in unhealthy dietary patterns like WD and the risk of developing different obstetric complications like hypertensive disorders of pregnancy [202], although other studies did not report any such association [203]. In general, 3 g to 5 g of daily sodium intake is recommended [204]. Previous works have evidenced that high-salt diets (more than 5 g of sodium per day) have detrimental effects on the placenta, altering its metabolism, function, inflammation, hypoxia, and other pathogenic processes [205,206]. Beauséjour et al. [207] also observed that pregnant Sprague–Dawley rats experienced a significant augmentation in oxidative stress markers in the placenta under increased sodium intake. Conversely, salt restriction in pregnancy also has negative implications in both the placenta and the fetus [208,209]. Collectively, these studies support the need for moderate sodium intake (less than 5 g/day), despite the need to ensure a minimum intake to prevent possible complications. Preventing the intake of UPFDs and a WD is the best advice to prevent excessive sodium intake, and the addition of salt to natural and non-processed foods might aid in ensuring the minimum required salt intake [113].

## 5. Conclusions

**Figure 3:**
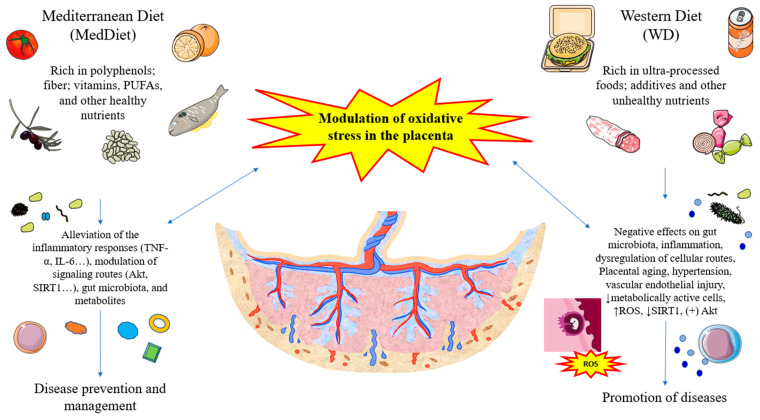
A summarized view of the role of different diets and their main nutrients in the modulation of oxidative stress in the placenta.

Oxidative stress in the placenta is a critical biological event involved in several physiological processes during pregnancy. However, exacerbated oxidative damage is associated with a plethora of diseases in pregnancy, like pre-eclampsia. As shown in Figure 3, different nutrients present in the diet, such as polyphenols, DF, omega 3, MUFAs, and vitamins, can favorably modulate oxidative stress in this organ, aiding in the prevention of different obstetric complications and the clinical management of different diseases. Conversely, compelling evidence shows that unhealthy dietary patterns like WDs and HFDs that include unbalanced nutrients and ultra-processed foods rich in additives and other components promote oxidative stress in the placenta. Although multiple studies have focused on the role of specific nutrients, mostly in in vitro animal models, further observational and intervention studies focusing on the placental structure and function in women with different dietary patterns should be encouraged to understand the precise influence of diet on this organ.

## Data Availability

No new data were created or analyzed in this study. Data sharing is not applicable to this article.
